# Heterotopic Pregnancy: Case Study and Literature Review in the Jordanian Royal Medical Services Context

**DOI:** 10.7759/cureus.99950

**Published:** 2025-12-23

**Authors:** Mothanna N Nawafleh, Salem M AL-Mousa, Abeer A AL-Smadi, Eman J AL-Omari, Marwan A AL-Shorman

**Affiliations:** 1 Obstetrics and Gynecology, Prince Rahid Bin Al-Hassan Military Hospital, Irbid, JOR

**Keywords:** diagnostic imaging, ectopic pregnancy, heterotopic pregnancy, laparoscopy, surgical management

## Abstract

Heterotopic pregnancy is a rare condition where intrauterine and extrauterine pregnancies occur simultaneously, posing significant diagnostic and management challenges. Timely recognition is critical, as it can lead to severe complications like rupture and hemorrhage and become a life-threatening condition if the ectopic component is not promptly addressed. This condition often requires careful monitoring and a tailored treatment approach to ensure the safety of both the mother and the intrauterine pregnancy. We reported a case of a 29-year-old woman, gravida 2para 1, who presented with lower abdominal pain in Prince Rashid Military Hospital/Jordanian Royal Medical Services, who was found to have a ruptured right tubal ectopic pregnancy with a significant amount of free fluid, despite having two intrauterine gestational sacs with viable fetuses. This case shows the significance of maintaining a high level of suspicion for heterotopic pregnancy. Recurrent imaging, symptom monitoring, and careful evaluation are essential for rapid diagnosis and decreasing maternal morbidity.

## Introduction

Heterotopic pregnancy is known as the simultaneous presence of an intrauterine pregnancy (IUP) and an ectopic pregnancy (EP) [1]. In naturally conceived pregnancies, this condition is considered extremely rare, with an estimated incidence ranging from 1 in 10,000 to 1 in 30,000 pregnancies [2]. The prevalence has increased significantly with the use of ovulation induction and assisted reproductive technologies (ART), reaching up to 1% in some ART-associated pregnancies [1].

Although advances in imaging and early pregnancy monitoring have occurred, heterotopic pregnancy remains a significant diagnostic challenge. Intrauterine pregnancy may create a false diagnosis and lead clinicians to overlook a concurrent ectopic gestation. A lot of patients remain asymptomatic during early stages, and others may present with nonspecific symptoms such as lower abdominal pain, adnexal tenderness, or vaginal bleeding. In more advanced cases, rupture of the ectopic component may result in acute abdomen, hemodynamic instability, or life-threatening intraperitoneal hemorrhage [3].

Early diagnosis of heterotopic pregnancy is critical to prevent serious maternal morbidity and mortality, as well as potential loss of the intrauterine pregnancy. Transvaginal ultrasonography plays a key role in diagnosis, but detection can be difficult, especially in early gestation or when imaging findings are unclear. Management strategies must be individualized, balancing maternal safety with preservation of the intrauterine pregnancy as much as possible [3]. This report describes a rare case of spontaneous heterotopic pregnancy diagnosed after tube rupture. We aim to show the importance of re-evaluation if symptoms remain; however, an IUP is confirmed. Reporting rare presentations of heterotopic pregnancy remains valuable to increase clinical awareness, especially in patients without traditional risk factors.

## Case presentation

A 29-year-old woman, who is para 1 (P1) from a previous cesarean section (C/S), had a history of secondary infertility for six years. After receiving ovulation induction drug (clomiphene citrate), she reported a regular menstrual cycle, with no history of abnormal vaginal bleeding. Her last menstrual period was reported to be about two months prior to presentation. She presented to the Obstetrics and Gynecology (OB/GYN) department emergency room with complaints of abdominal pain that had started six hours prior to presentation, nausea, vomiting, and shoulder pain. On first evaluation, she was hemodynamically stable, with a blood pressure of 127/81 mmHg, a pulse rate was 85 beats/min, and body temperature was 37.2°C.

**Figure 1:**
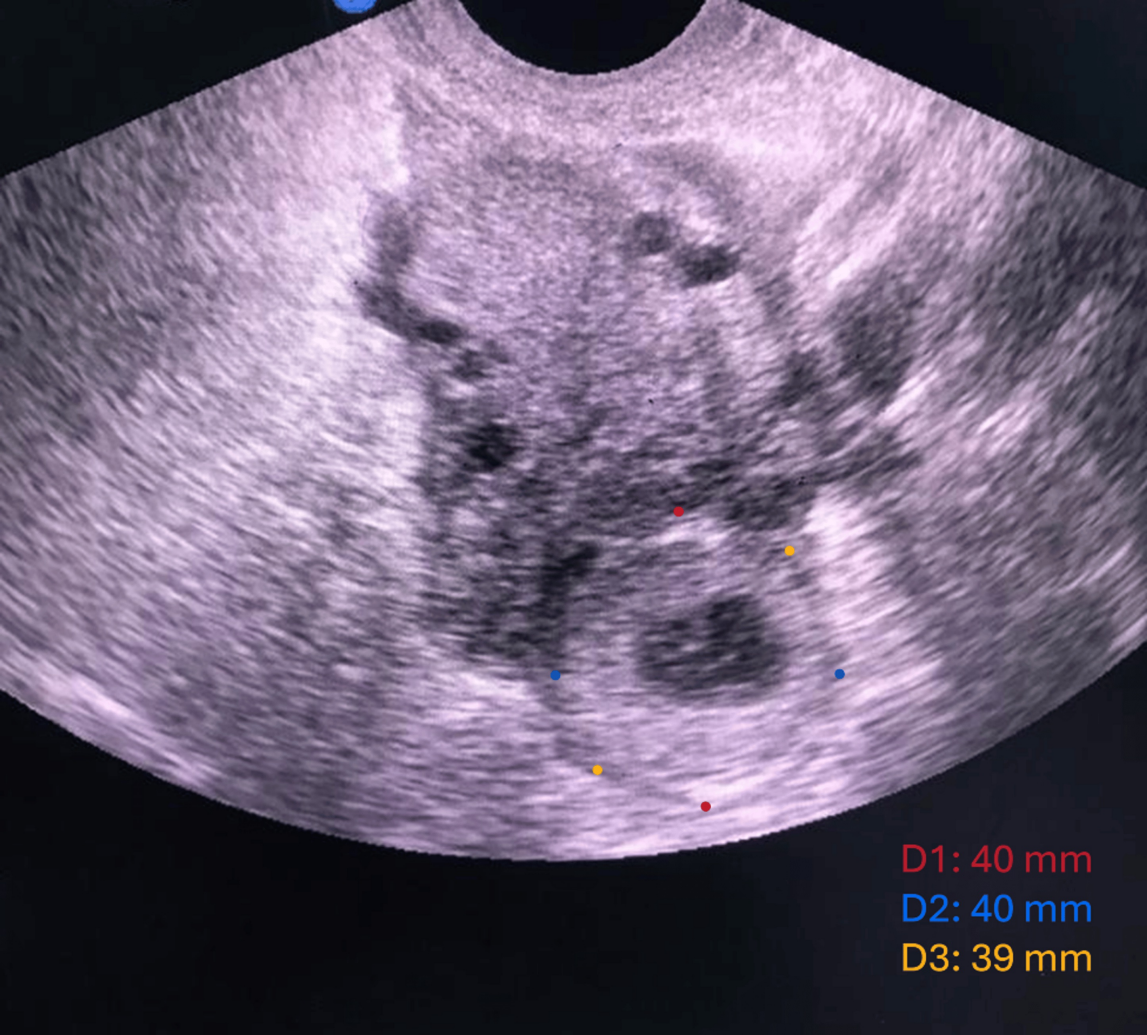
Transvaginal ultrasound shows right adnexal mass.

**Figure 2:**
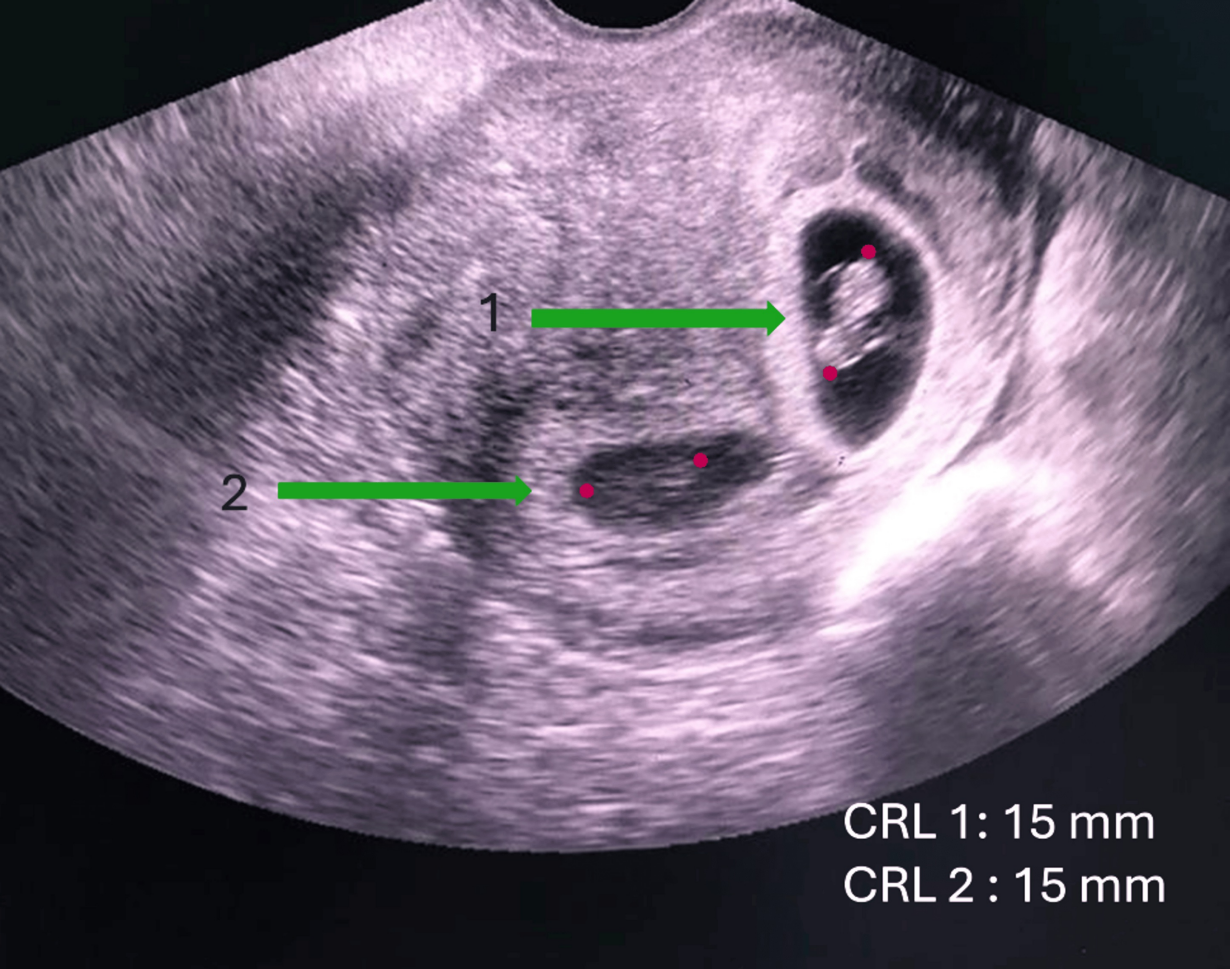
Transvaginal ultrasound shows the presence of two intrauterine gestational sacs with positive fetal echoes.

A transvaginal scan revealed two intrauterine gestational sacs with viable fetuses, with a crown-rump length (CRL) measuring 15 mm for each one, corresponding to approximately seven weeks of gestation. Additionally, a 4x4 x3.9 cm right adnexal mass and free fluid in the pouch of Douglas (POD) were observed (Figures 1, 2).

**Figure 3:**
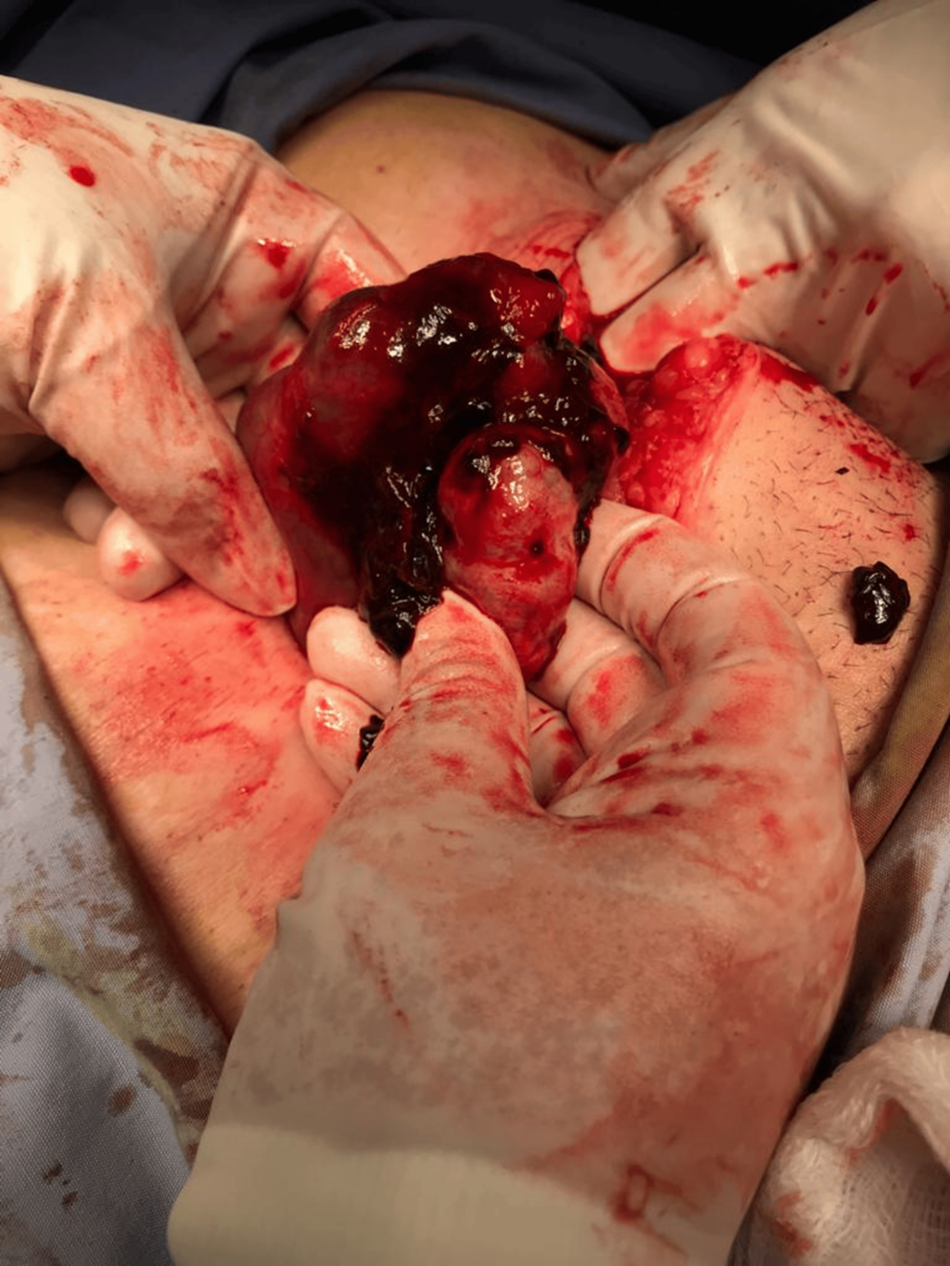
Ruptured right tubal ectopic pregnancy.

**Figure 4:**
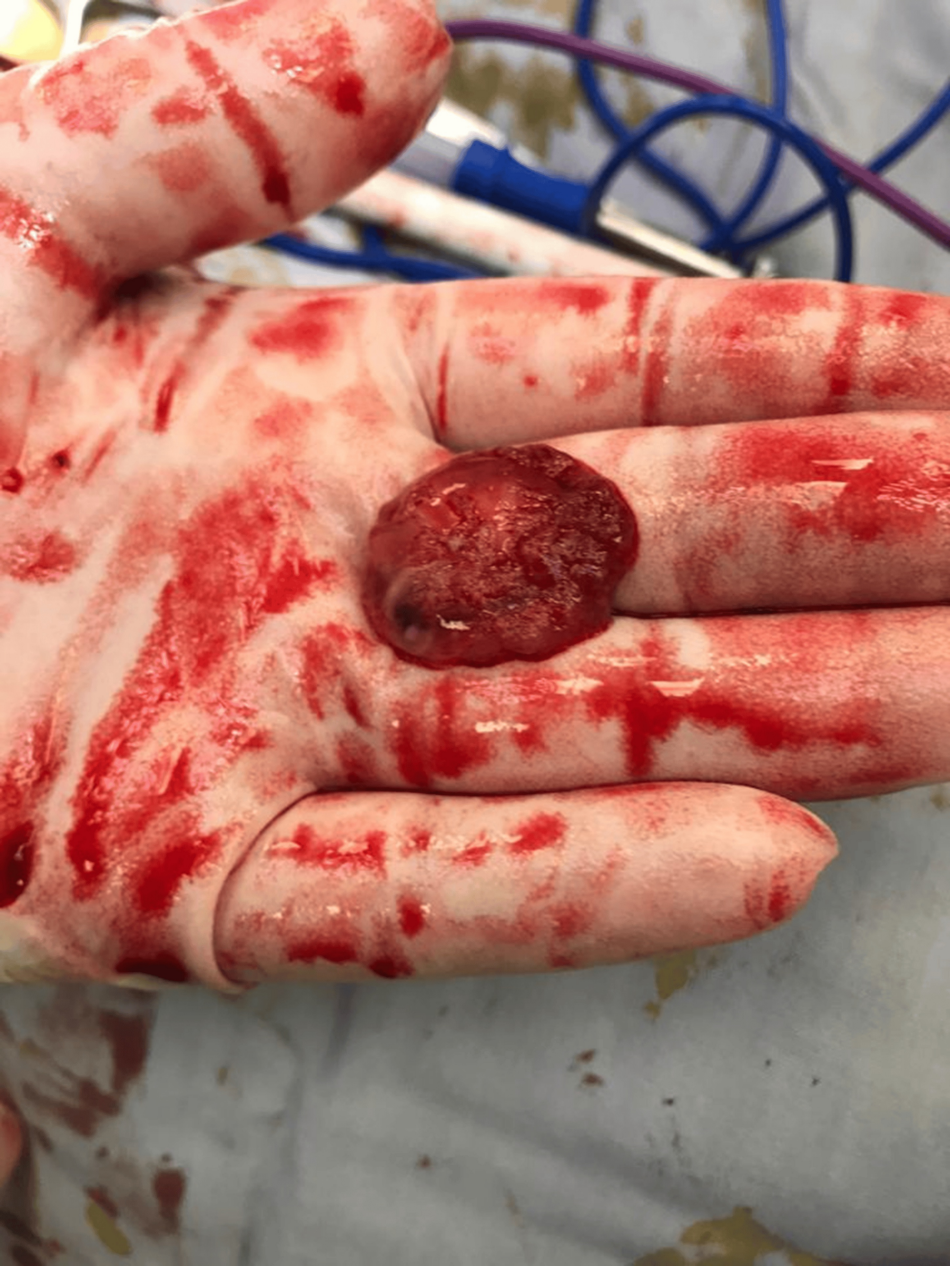
Ectopic specimen.

The differential diagnosis included a complicated hemorrhagic ovarian cyst, acute appendicitis, or a coexisting ectopic pregnancy. Her symptoms deteriorated progressively during observation, with worsening abdominal pain and increasing adnexal tenderness, and she became hemodynamically unstable; urgent laparotomy was performed for evaluation. The results indicated a ruptured right tubal ectopic pregnancy with a significant amount of free fluid, about 700 ml. An emergency right salpingectomy was conducted, with careful preservation of the intrauterine pregnancy (Figures 3, 4).

The postoperative course was uneventful. Pathological examination of the surgical specimen confirmed the diagnosis of a ruptured ectopic pregnancy. Meanwhile, the intrauterine pregnancy continued without complications. At 37 weeks of gestation, the patient went into labor and underwent a cesarean delivery. The outcome was the birth of two healthy infants: a male weighing 2.150 kg and a female weighing 2.500 kg, with no need for ICU admission.

## Discussion

Heterotopic pregnancy presents a significant diagnostic challenge due to its rare occurrence and the simultaneous presence of both intrauterine and ectopic gestations [4]. This complexity is compounded by the fact that the symptoms of ectopic pregnancy-such as abdominal pain, vaginal bleeding, and hemodynamic instability often masked by the ongoing intrauterine pregnancy [5]. In the case presented, the patient's history of secondary infertility, use of ovulation induction drugs, and the subsequent development of heterotopic pregnancy highlight the increasing incidence of this condition in the context of assisted reproductive technologies.

A high index of suspicion facilitated the diagnosis of heterotopic pregnancy in this patient, given her clinical presentation and risk factors. The transvaginal ultrasound was pivotal in identifying both intrauterine and ectopic pregnancies, reinforcing the critical role of imaging in the early diagnosis of this condition. The detection of a right adnexal mass and the presence of free fluid in the pouch of Douglas, along with the ultrasound findings of two viable intrauterine gestational sacs, pointed toward the coexistence of an ectopic pregnancy, which was later confirmed intraoperatively.

Therapeutic management in this case required immediate surgical intervention due to the patient's hemodynamic instability caused by the ruptured right tubal ectopic pregnancy. The decision to perform a right salpingectomy while preserving the intrauterine pregnancy underscores the delicate balance required in managing such cases [6]. The successful preservation of the intrauterine pregnancy, which progressed uneventfully to term, illustrates the potential for favorable outcomes with timely and appropriate surgical intervention [7].

This case also emphasizes the importance of multidisciplinary care and vigilant postoperative monitoring. The patient’s postoperative course was uneventful, and the continued viability of the intrauterine pregnancy was confirmed through regular follow-up. The eventual delivery of healthy twins via cesarean section further underscores the effectiveness of the treatment strategy employed.

The importance of early diagnosis cannot be overstated, as delayed recognition of a heterotopic pregnancy could have led to more severe maternal complications [8], including rupture and hemorrhage, which could compromise both maternal and fetal outcomes [8]. The use of transvaginal ultrasonography remains the gold standard for early diagnosis, but it is crucial to maintain a high index of suspicion, particularly in patients undergoing assisted reproductive technologies [9]. Additionally, the presence of nonspecific symptoms in heterotopic pregnancy cases highlights the necessity for clinicians to be thorough in their evaluations to avoid misdiagnosis [10].

In reviewing the literature, it is evident that heterotopic pregnancies are becoming more frequently diagnosed due to the widespread use of assisted reproductive technologies [10]. However, despite advances in diagnostic modalities, the condition remains challenging due to its rarity and the nonspecific nature of its symptoms. The literature also highlights the variability in treatment approaches, ranging from expectant management to surgical intervention, depending on the clinical scenario [11,12].

Furthermore, this case underscores the importance of considering fertility preservation in the management of heterotopic pregnancies. The successful continuation of the intrauterine pregnancy, resulting in the delivery of healthy twins, exemplifies the positive maternal and neonatal outcomes that can be achieved with precise and timely surgical intervention [13]. These findings support the consideration of a fertility-sparing approach when feasible, as preserving the intrauterine pregnancy in cases like this can optimize long-term reproductive outcomes [13].

Future research should focus on the development of standardized diagnostic and management protocols for heterotopic pregnancy to further improve outcomes. Additionally, exploring the potential of non-surgical management in early diagnosed stable cases could provide alternative therapeutic options that preserve fertility and reduce morbidity. The integration of these strategies into clinical practice could enhance the care provided to patients with this complex obstetric condition, ultimately improving both maternal and fetal outcomes.

Lastly, this case highlights the growing need for tailored clinical guidelines, particularly in regions where assisted reproductive technologies are increasing. As more patients undergo fertility treatments, heterotopic pregnancies may become more prevalent. Developing specific protocols for managing such cases will help reduce maternal morbidity and improve neonatal outcomes, ensuring that both the intrauterine and ectopic pregnancies are appropriately addressed.

## Conclusions

Heterotopic pregnancy represents a diagnostic and therapeutic challenge, particularly in settings where assisted reproductive technologies are utilized. This case underscores the critical need for early detection and appropriate management strategies to optimize maternal and fetal outcomes. The preservation of intrauterine pregnancies, even in the context of an ectopic rupture, is achievable with timely intervention and meticulous surgical technique.
